# Supplementary material for: Influence of patient isolation due to colonization with multidrug-resistant organisms on functional recovery after spinal cord injury
Source: PLoS One. 2021 Mar 26;16(3):e0249295. doi: 10.1371/journal.pone.0249295 (PMC7997009; doi:10.1371/journal.pone.0249295)
Supplement: S1 Table — (DOCX) [file pone.0249295.s001.docx]

S1 Table: Neurological and functional status of matched participants at early stage.

| **No** | **Sex** | **AIS** | **NLI** | **SCIM** | **UEMS** | **LEMS** | **MDRO status** |
| --- | --- | --- | --- | --- | --- | --- | --- |
| 1 | m | A | T4 | 22 | 50 | 0 | 4-MRGN |
| 2 | m | A | T1 | 13 | 50 | 0 | negative |
| 3 | m | C | C2 | 5 | 12 | 29 | MRSA |
| 4 | f | C | C4 | 10 | 12 | 32 | negative |
| 5 | f | A | C3 | 0 | 14 | 0 | 4-MRGN |
| 6 | m | A | C2 | 0 | 14 | 0 | negative |
| 7 | m | C | T4 | 17 | 50 | 11 | 4-MRGN |
| 8 | f | D | C4 | 23 | 50 | 10 | negative |
| 9 | f | B | T2 | 12 | 50 | 0 | MRSA |
| 10 | m | A | T2 | 11 | 50 | 0 | negative |
| 11 | m | B | C5 | 14 | 25 | 0 | MRSA |
| 12 | m | A | C4 | 10 | 28 | 0 | negative |
| 13 | f | C | C6 | 13 | 27 | 14 | 4-MRGN |
| 14 | m | C | C5 | 19 | 30 | 11 | negative |
| 15 | m | A | T4 | 0 | 50 | 0 | MRSA |
| 16 | f | A | NA | 0 | 50 | 0 | negative |
| 17 | f | A | C5 | 0 | 14 | 0 | MRSA |
| 18 | f | B | C5 | 0 | 11 | 0 | negative |
| 19 | m | A | C4 | 0 | 19 | 0 | MRSA |
| 20 | m | C | C3 | 2 | 18 | 0 | negative |
| 21 | m | A | NA | 0 | 2 | 8 | 4-MRGN |
| 22 | f | C | C2 | 0 | 3 | 0 | negative |
| 23 | m | C | C2 | 10 | 11 | 21 | MRSA |
| 24 | m | C | C4 | 18 | 14 | 26 | negative |
| 25 | m | A | C2 | 0 | 6 | 0 | MRSA |
| 26 | m | A | C4 | 2 | 4 | 0 | negative |
| **Mean group** |  |  |  | **7** | **25** | **6** | **MDRO-positive** |
| **Mean group** |  |  |  | **8** | **26** | **6** | **MDRO-negative** |
