# Supplementary material for: Influence of patient isolation due to colonization with multidrug-resistant organisms on functional recovery after spinal cord injury
Source: PLoS One. 2021 Mar 26;16(3):e0249295. doi: 10.1371/journal.pone.0249295 (PMC7997009; doi:10.1371/journal.pone.0249295)
Supplement: S2 Table — (DOCX) [file pone.0249295.s002.docx]

**S2 Table: SCIM and WISCI II of matched incomplete patients at late stage**

| No | SCIM | SCIM Mobility | WISCI II | Group |
| --- | --- | --- | --- | --- |
| 3 | 55 | 16 | 0 | MDRO-positive |
| 4 | 27 | 9 | 8 | MDRO-negative |
| 7 | 64 | 17 | 1 | MDRO-positive |
| 8 | 71 | 16 | 0 | MDRO-negative |
| 13 | 69 | 17 | 4 | MDRO-positive |
| 14 | 50 | 15 | 0 | MDRO-negative |
| 23 | 26 | 7 | 0 | MDRO-positive |
| 24 | 91 | 40 | 20 | MDRO-negative |
